# Aggressive Tumor of the Midface

**Published:** 2014-08-21

**Authors:** Adrian Frunza, Dragos Slavescu, Ioan Lascar

**Affiliations:** Bucharest Emergency Clinical Hospital, Bucharest University School of Medicine, Romania

**Keywords:** midface tumor, basal cell carcinoma, cheek defects, frontal flap, face reconstruction

**Figure:**
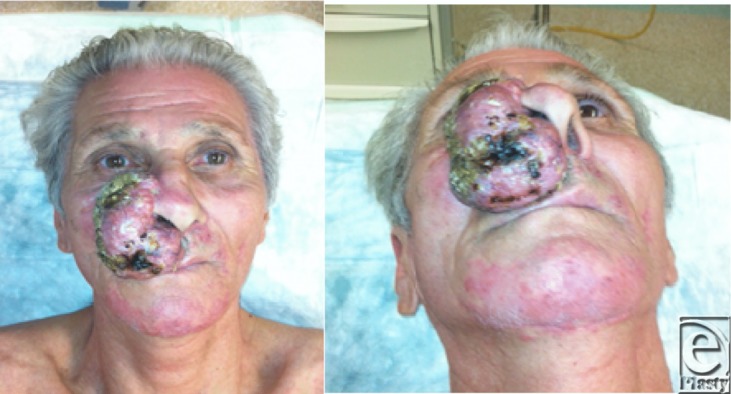
Preoperative photographs

## DESCRIPTION

A 63-year-old man came in our clinic with a giant basal cell tumor, which had appeared 6 years ago. The computed tomographic scan showed no infiltration of the underlying bone; no intracranial tumoral extensions. The excisional defect was covered by a frontal flap and a cheek advancement flap.

## QUESTIONS

1. **What is the most frequent malignant epithelial tumor of the face?**
2. **Discuss the differential diagnosis of this lesion.**
3. **What are the surgical options for tumor removal and reconstruction?**
4. **What alternative treatments other than surgical excision are available for basal cell carcinoma (BCC)?**

## DISCUSSION

Basal cell carcinoma is the most frequent malignant epithelial tumor of the face.^1^ Male patients are affected twice as often as women; the risk of developing BCC increases with age and is related to chronic exposure to the sun. Other risk factors are X-ray exposure, arsenic exposure, associated conditions like xeroderma pigmentosum, basal cell nevus syndrome (Gorlin-Goltz syndrome), etc.^2^ Basal cell carcinoma is a local invasive tumor which develops finger-like outgrowths, destroying the surrounding tissues. Metastases are rare. Among histological subtypes, we encounter the superficial, nodular, pigmented, morphoeic, and infiltrative forms. The basosquamous form is a particular variant, very aggressive in terms of both local and systemic extensions.^3^

The differential diagnosis entails the following: actinic keratosis, melanocytic nevi, squamous cell carcinoma, malignant melanoma, angiofibroma, sebaceous hyperplasia, Merkel cell carcinoma, trichoepithelioma/trichoblastoma, and microcystic adnexal carcinoma. It is of paramount importance that a local biopsy is performed to have the necessary preliminary data and to decide the appropriate treatment or if a neoadjuvant therapy would be necessary. In our case, a punch biopsy confirmed the clinical suspicion of BCC.

In the majority of cases, surgery is the recommended treatment, especially for large tumors. The surgical margins of the excisional specimen should be carefully evaluated by a pathologist for presence of the characteristic stroma; if seen, recurrence is possible and reexcision has to be performed. Adequate excision nearly always leads to cure. For a small tumor (<20 mm in diameter), a 3-mm peripheral surgical margin usually leads to cure; for large, recurrent tumors or aggressive types (morphoeic BCC), the necessary surgical margin is wider, about 5 to 10 mm.^4^ The subsequent facial defect must be considered as a 3-dimensional structure to be covered with similar tissues (in terms of structure, color, and texture). The goal is to reconstruct the facial aesthetic units as good as possible. The reconstructive options range from the classical skin grafts and local transposition flaps to free flaps (for this patient, a radial or a dorsalis pedis free flap). In the presented case, we have chosen the use of local flaps (a cheek advancement flap and a frontal flap folded on itself at the tip) given the nasal full-thickness defect associated with the cheek partial-thickness defect. This option has led to fast local healing and avoided the need to cover any secondary defect by other methods (skin grafts). The use of flaps also ensured local stability with further radiotherapy. The patient age, lifestyle, and comorbidities (diabetes mellitus, smoker) also influenced our decision.

Other available options are Mohs micrographic surgery, curettage, electrodesiccation and cautery, cryotherapy. US Food and Drug Administration approved the use of imiquimod (Aldara) for superficial BCCs less than 2 cm in diameter. Aldara is an immune-response modifier whose action is to promote both innate and adaptive cell-mediated immune responses. It is applied daily, 5 days a week for 6 to 12 weeks. Another topical treatment is using fluorouracil administered every 12 hours for 3 to 6 weeks.^5^ Interferon alfa has been used with success for small, nodular, and superficial BCCs by intralesional injections.^6^ For recurrent tumors, radiotherapy is a suitable choice. It may also be used for difficult primary lesions.^7^ As a palliative treatment, we can also use photodynamic therapy.^8^ This is done by administering orally, parenterally, or topically a tumor sensitizer (5-aminolevulinic acid) activated by light. The choice of the therapeutic method must consider any coexisting medical conditions and age. Sometimes an aggressive treatment will cause more problems than the tumor itself. However, surgical excision is the best choice for a big tumoral mass. The other methods described earlier are suited for small and superficial lesions. They also have the disadvantage of lacking the pathologic examination. Following treatment, there is a risk of tumor local recurrence (incompletely excised lesions); a new tumor may appear as well. These patients must be educated for self-monitoring or follow-up in a primary care setting.

In summary, many treatment options are available for a given tumor. There are minimal invasive modalities and more destructive ones. The tumor recurrence risk (increases with aggressive histological subtypes and dimensions) will lead to the appropriate method of treatment.

**Figure:**
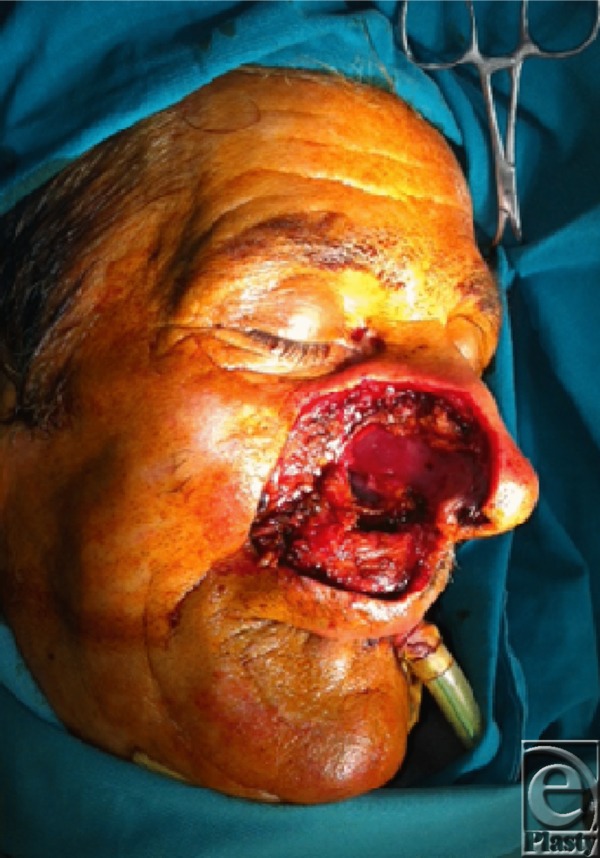
The facial defect after excision

**Figure:**
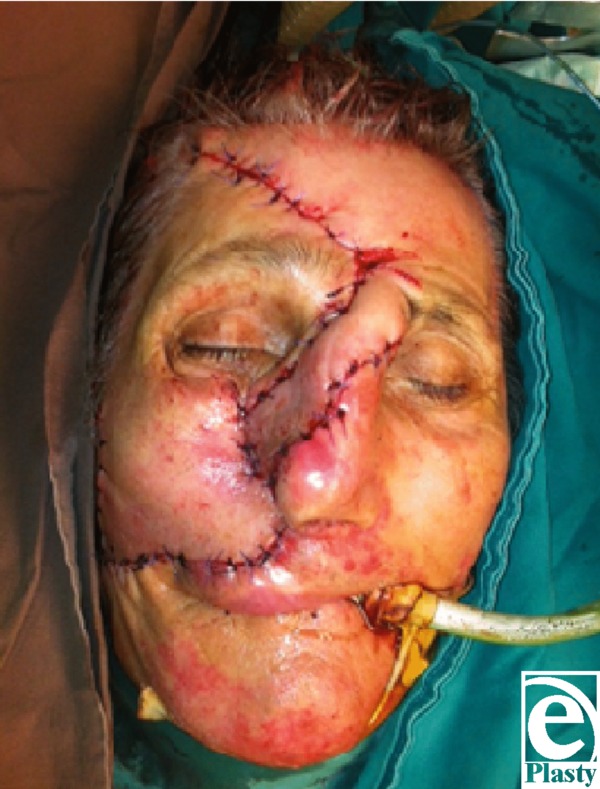
Immediate postoperative aspect

**Figure:**
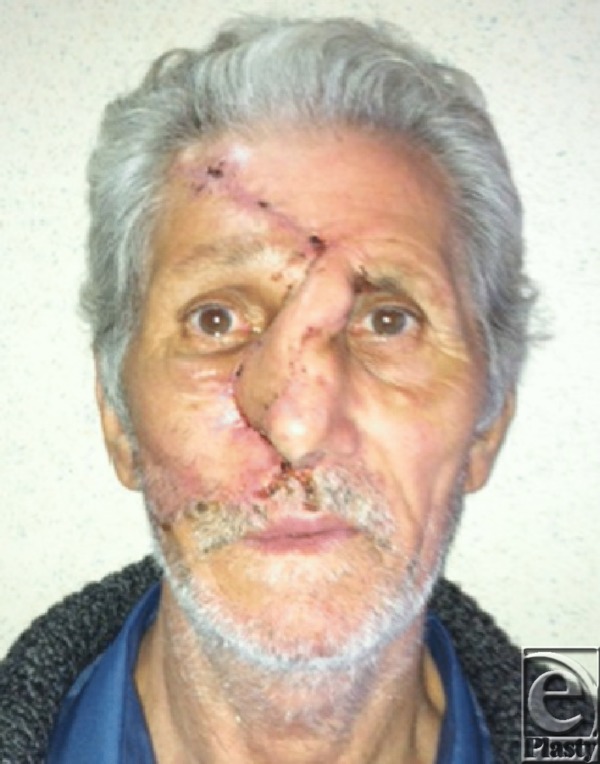
12 days postoperative aspect
